# 6-Bromo­imidazo[1,2-*a*]pyridin-8-amine

**DOI:** 10.1107/S1600536811040177

**Published:** 2011-10-05

**Authors:** Siham Dahmani, Youssef Kandri Rodi, Frederic Capet, El Mokhtar Essassi, Seik Weng Ng

**Affiliations:** a Laboratoire de Chimie Organique Appliquée, Faculté des Sciences et Techniques, Université Sidi Mohamed Ben Abdallah, Fés, Morocco; b Unité de Catalyse et de Chimie du Solide, Ecole Nationale Supérieure de Chimie de Lille, Lille, France; c Laboratoire de Chimie Organique Hétérocyclique, Pôle de Compétences Pharmacochimie, Université Mohammed V-Agdal, BP 1014 Avenue Ibn Batout, Rabat, Morocco; d Department of Chemistry, University of Malaya, 50603 Kuala Lumpur, Malaysia; e Chemistry Department, King Abdulaziz University, PO Box 80203 Jeddah, Saudi Arabia

## Abstract

The title compound, C_7_H_6_BrN_3_, crystallizes with three independent mol­ecules in the asymmetric unit. The mol­ecules are approximately planar (r.m.s. deviations for all non-H atoms = 0.016, 0.023 and 0.024 Å). The primary amine groups show pyramidal coordination. In the crystal, adjacent mol­ecules are linked by N—H⋯N hydrogen bonds. For two independent mol­ecules, the amine groups are hydrogen-bond donors *via* one H atom to one acceptor atom, whereas for the third independent mol­ecule, the amine group is a hydrogen-bond donor to two acceptor atoms.

## Related literature

For background information on 8-amino-6-bromo-imidazo[1,2-*a*]pyridine, see: Dwyer *et al.* (2007 ▶).
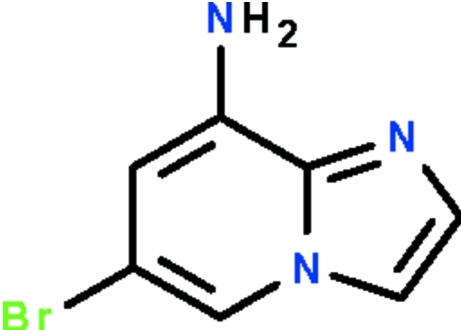


## Experimental

### 

#### Crystal data

C_7_H_6_BrN_3_
*M* *_r_* = 212.06
Monoclinic, 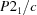

*a* = 15.1378 (5) Å
*b* = 21.2006 (8) Å
*c* = 6.9744 (3) Å
β = 92.6106 (7)°
*V* = 2235.97 (15) Å^3^
*Z* = 12
Mo *K*α radiation
μ = 5.44 mm^−1^
*T* = 100 K
0.13 × 0.04 × 0.02 mm

#### Data collection

Bruker APEX DUO diffractometer
Absorption correction: multi-scan *SADABS* (Sheldrick, 1996 ▶)’ *T*_min_ = 0.538, *T*_max_ = 0.899
44729 measured reflections
5538 independent reflections
4675 reflections with *I* > 2σ(*I*)
*R*_int_ = 0.048

#### Refinement

*R*[*F* ^2^ > 2σ(*F* ^2^)] = 0.031
*wR*(*F* ^2^) = 0.078
*S* = 1.08
5538 reflections
322 parameters
6 restraints
H atoms treated by a mixture of independent and constrained refinement
Δρ_max_ = 1.15 e Å^−3^
Δρ_min_ = −0.46 e Å^−3^

### 

Data collection: *APEX2* (Bruker, 2009 ▶); cell refinement: *SAINT* (Bruker, 2009 ▶); data reduction: *SAINT*; program(s) used to solve structure: *SHELXS97* (Sheldrick, 2008 ▶); program(s) used to refine structure: *SHELXL97* (Sheldrick, 2008 ▶); molecular graphics: *X-SEED* (Barbour, 2001 ▶); software used to prepare material for publication: *publCIF* (Westrip, 2010 ▶).

## Supplementary Material

Crystal structure: contains datablock(s) global, I. DOI: 10.1107/S1600536811040177/bt5659sup1.cif

Structure factors: contains datablock(s) I. DOI: 10.1107/S1600536811040177/bt5659Isup2.hkl

Supplementary material file. DOI: 10.1107/S1600536811040177/bt5659Isup3.cml

Additional supplementary materials:  crystallographic information; 3D view; checkCIF report

## Figures and Tables

**Table 1:** Hydrogen-bond geometry (Å, °)

*D*—H⋯*A*	*D*—H	H⋯*A*	*D*⋯*A*	*D*—H⋯*A*
N3—H31⋯N5	0.88 (1)	2.16 (1)	3.034 (3)	169 (4)
N6—H61⋯N2	0.88 (1)	2.23 (1)	3.094 (3)	168 (3)
N9—H91⋯N8^i^	0.87 (1)	2.27 (2)	3.091 (3)	158 (3)
N9—H92⋯N6^ii^	0.88 (1)	2.27 (1)	3.143 (3)	177 (3)

Symmetry codes: (i) 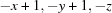; (ii) 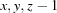.

**Fig. 1.**
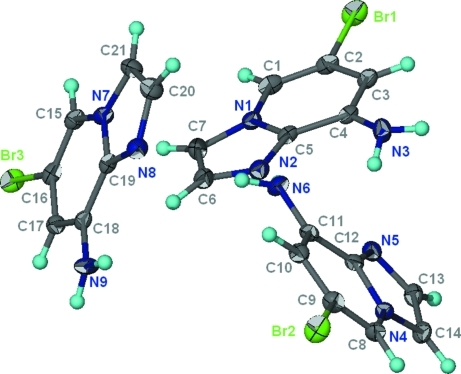
Anisotropic displacement ellipsoid plot (Barbour, 2001) of the three independent molecules at the 70% probability level; hydrogen atoms are drawn as spheres of arbitrary radius.

## supplementary crystallographic information

### Comment

8-Amino-6-bromo-imidazo[1,2-*a*]pyridine (Scheme I) was recently evaluated for its as a cyclin-dependent kinase-2 (CDK2) inhibitor (Dwyer *et al.*, 2007). We have synthesized the compound for use in a similar study. The non-H atoms of the three independent molecules of C_7_H_6_BrN_3_ are planar (Fig. 1). Their primary amino groups show pyramidal coordination, and adjacent molecules are linked by N–H···N hydrogen bonds to form a layer structure. For two independent molecules, their amino groups are each a hydrogen-bond donor to one acceptor atom whereas for the third independent molecule, its amino group is hydrogen-bond bond to two acceptor atoms (Table 1).

### Experimental

A mixture of chloroacetaldehyde (0.68 ml, 10.6 mmol), 5-bromo-2,3-diaminopyridine (1 g, 5.32 mmol) and sodium bicarbonate (0.44 g, 5.32 mmol) in ethanol (10 ml) was heated. The reaction was monitored by TLC. On completion of the reaction, the solution was extracted with dichloromethane and the organic layer was dried over anhydrous sodium sulfate. Evaporation of the solvent followed by recrystallization from hexane gave yellow crystals of the title compound.

### Refinement

Carbon-bound H-atoms were placed in calculated positions (C– 0.95 Å) and were included in the refinement in the riding model approximation, with *U*(H) set to 1.2*U*(C).

The amino H-atoms were located in a difference Fourier map, and were refined isotropically with a distance restraint of N–H 0.88±0.01 Å.

The final difference Fourier map had a peak in the vicinity of Br1 and a hole in the vicinity of Br2.

### Crystal data

**Table:**

C_7_H_6_BrN_3_	*F*(000) = 1248
*M**_r_* = 212.06	*D*_x_ = 1.890 Mg m^−^^3^
Monoclinic, *P*2_1_/*c*	Mo *K*α radiation, λ = 0.71073 Å
Hall symbol: -P 2ybc	Cell parameters from 9878 reflections
*a* = 15.1378 (5) Å	θ = 2.4–28.2°
*b* = 21.2006 (8) Å	µ = 5.44 mm^−^^1^
*c* = 6.9744 (3) Å	*T* = 100 K
β = 92.6106 (7)°	Prism, yellow
*V* = 2235.97 (15) Å^3^	0.13 × 0.04 × 0.02 mm
*Z* = 12	

### Data collection

**Table:**

Bruker APEX DUO diffractometer	5538 independent reflections
Radiation source: fine-focus sealed tube	4675 reflections with *I* > 2σ(*I*)
graphite	*R*_int_ = 0.048
ω scans	θ_max_ = 28.3°, θ_min_ = 1.7°
Absorption correction: multi-scan *SADABS* (Sheldrick, 1996)'	*h* = −20→20
*T*_min_ = 0.538, *T*_max_ = 0.899	*k* = −28→28
44729 measured reflections	*l* = −9→9

### Refinement

**Table:**

Refinement on *F*^2^	Primary atom site location: structure-invariant direct methods
Least-squares matrix: full	Secondary atom site location: difference Fourier map
*R*[*F*^2^ > 2σ(*F*^2^)] = 0.031	Hydrogen site location: inferred from neighbouring sites
*wR*(*F*^2^) = 0.078	H atoms treated by a mixture of independent and constrained refinement
*S* = 1.08	*w* = 1/[σ^2^(*F*_o_^2^) + (0.0398*P*)^2^ + 2.2806*P*] where *P* = (*F*_o_^2^ + 2*F*_c_^2^)/3
5538 reflections	(Δ/σ)_max_ = 0.001
322 parameters	Δρ_max_ = 1.15 e Å^−^^3^
6 restraints	Δρ_min_ = −0.46 e Å^−^^3^

### Fractional atomic coordinates and isotropic or equivalent isotropic displacement parameters (Å^2^)

**Table:**

	*x*	*y*	*z*	*U*_iso_*/*U*_eq_	
Br1	1.107067 (18)	0.309517 (13)	0.81184 (4)	0.02396 (8)	
Br2	0.248212 (17)	0.345125 (13)	0.22269 (4)	0.02332 (8)	
Br3	0.930771 (17)	0.485358 (12)	−0.30860 (4)	0.01955 (7)	
N1	0.93022 (14)	0.35309 (10)	0.3739 (3)	0.0147 (4)	
N2	0.78304 (15)	0.35253 (10)	0.3158 (3)	0.0170 (4)	
N3	0.76115 (15)	0.29900 (11)	0.6916 (3)	0.0179 (4)	
N4	0.46555 (13)	0.23784 (10)	0.3560 (3)	0.0131 (4)	
N5	0.61032 (14)	0.25193 (10)	0.4281 (3)	0.0153 (4)	
N6	0.58767 (15)	0.38763 (10)	0.3737 (3)	0.0167 (4)	
N7	0.75458 (14)	0.52802 (10)	0.0966 (3)	0.0144 (4)	
N8	0.60864 (14)	0.52091 (10)	0.1372 (3)	0.0165 (4)	
N9	0.58646 (14)	0.46594 (10)	−0.2438 (3)	0.0164 (4)	
C1	1.00873 (17)	0.34402 (11)	0.4792 (4)	0.0174 (5)	
H1	1.0643	0.3540	0.4290	0.021*	
C2	1.00202 (17)	0.32018 (12)	0.6573 (4)	0.0168 (5)	
C3	0.92127 (17)	0.30293 (11)	0.7364 (4)	0.0167 (5)	
H3	0.9207	0.2850	0.8612	0.020*	
C4	0.84294 (17)	0.31237 (11)	0.6302 (4)	0.0145 (5)	
C5	0.84843 (16)	0.33958 (11)	0.4443 (4)	0.0142 (5)	
C6	0.82481 (18)	0.37419 (12)	0.1590 (4)	0.0196 (5)	
H6	0.7949	0.3870	0.0427	0.023*	
C7	0.91483 (18)	0.37524 (13)	0.1893 (4)	0.0194 (5)	
H7	0.9575	0.3884	0.1020	0.023*	
C8	0.37957 (17)	0.25553 (12)	0.3112 (4)	0.0158 (5)	
H8	0.3333	0.2254	0.2961	0.019*	
C9	0.36455 (17)	0.31802 (12)	0.2898 (4)	0.0162 (5)	
C10	0.43202 (17)	0.36433 (12)	0.3074 (4)	0.0157 (5)	
H10	0.4183	0.4076	0.2870	0.019*	
C11	0.51705 (17)	0.34614 (11)	0.3540 (3)	0.0144 (5)	
C12	0.53420 (16)	0.28070 (11)	0.3834 (3)	0.0134 (5)	
C13	0.58955 (18)	0.18878 (12)	0.4262 (4)	0.0173 (5)	
H13	0.6312	0.1560	0.4528	0.021*	
C14	0.50220 (18)	0.17886 (12)	0.3818 (4)	0.0171 (5)	
H14	0.4726	0.1394	0.3708	0.021*	
C15	0.83221 (16)	0.52183 (11)	0.0020 (4)	0.0154 (5)	
H15	0.8873	0.5358	0.0574	0.018*	
C16	0.82568 (16)	0.49497 (11)	−0.1733 (4)	0.0152 (5)	
C17	0.74557 (16)	0.47370 (11)	−0.2611 (4)	0.0142 (5)	
H17	0.7450	0.4540	−0.3835	0.017*	
C18	0.66754 (16)	0.48172 (11)	−0.1679 (4)	0.0135 (5)	
C19	0.67320 (16)	0.50949 (11)	0.0182 (4)	0.0131 (5)	
C20	0.65091 (18)	0.54752 (13)	0.2963 (4)	0.0206 (5)	
H20	0.6217	0.5610	0.4069	0.025*	
C21	0.73987 (18)	0.55214 (13)	0.2758 (4)	0.0183 (5)	
H21	0.7827	0.5686	0.3662	0.022*	
H31	0.7175 (18)	0.2903 (18)	0.608 (4)	0.045 (11)*	
H32	0.755 (3)	0.2726 (15)	0.787 (4)	0.047 (12)*	
H61	0.6413 (10)	0.3726 (14)	0.367 (4)	0.021 (8)*	
H62	0.581 (2)	0.4227 (9)	0.307 (4)	0.019 (8)*	
H91	0.5382 (13)	0.4706 (14)	−0.181 (4)	0.018 (8)*	
H92	0.586 (2)	0.4427 (14)	−0.348 (3)	0.033 (10)*	

### Atomic displacement parameters (Å^2^)

**Table:**

	*U*^11^	*U*^22^	*U*^33^	*U*^12^	*U*^13^	*U*^23^
Br1	0.01367 (13)	0.02313 (14)	0.03436 (16)	−0.00028 (10)	−0.00679 (11)	0.00462 (11)
Br2	0.01282 (13)	0.02352 (14)	0.03321 (16)	0.00291 (10)	−0.00335 (10)	0.00457 (11)
Br3	0.01248 (12)	0.02091 (13)	0.02567 (14)	−0.00039 (9)	0.00531 (9)	−0.00224 (10)
N1	0.0135 (10)	0.0149 (10)	0.0158 (10)	−0.0029 (8)	0.0020 (8)	−0.0011 (8)
N2	0.0168 (10)	0.0159 (10)	0.0182 (11)	−0.0023 (8)	−0.0023 (8)	0.0018 (8)
N3	0.0138 (11)	0.0231 (12)	0.0167 (11)	−0.0022 (9)	0.0012 (8)	0.0053 (9)
N4	0.0119 (10)	0.0138 (10)	0.0135 (10)	0.0000 (8)	−0.0009 (8)	0.0002 (8)
N5	0.0155 (10)	0.0162 (10)	0.0142 (10)	0.0002 (8)	−0.0006 (8)	0.0010 (8)
N6	0.0151 (11)	0.0132 (10)	0.0215 (11)	−0.0021 (8)	−0.0002 (9)	−0.0003 (8)
N7	0.0110 (10)	0.0150 (10)	0.0171 (10)	−0.0012 (8)	−0.0015 (8)	−0.0009 (8)
N8	0.0155 (10)	0.0158 (10)	0.0183 (11)	0.0003 (8)	0.0021 (8)	−0.0005 (8)
N9	0.0117 (10)	0.0190 (11)	0.0184 (11)	−0.0017 (8)	−0.0014 (8)	−0.0056 (9)
C1	0.0107 (11)	0.0159 (12)	0.0255 (14)	−0.0003 (9)	0.0009 (10)	−0.0034 (10)
C2	0.0121 (11)	0.0141 (11)	0.0240 (13)	0.0007 (9)	−0.0028 (10)	−0.0009 (10)
C3	0.0178 (13)	0.0122 (11)	0.0201 (13)	−0.0009 (9)	−0.0005 (10)	0.0007 (9)
C4	0.0140 (12)	0.0105 (10)	0.0190 (12)	−0.0007 (9)	0.0018 (9)	−0.0007 (9)
C5	0.0122 (11)	0.0119 (11)	0.0187 (12)	−0.0014 (9)	0.0021 (9)	−0.0018 (9)
C6	0.0210 (13)	0.0185 (12)	0.0188 (13)	−0.0054 (10)	−0.0037 (10)	0.0033 (10)
C7	0.0224 (13)	0.0198 (12)	0.0162 (12)	−0.0052 (10)	0.0022 (10)	0.0023 (10)
C8	0.0140 (12)	0.0176 (12)	0.0156 (12)	−0.0023 (9)	−0.0016 (9)	0.0005 (9)
C9	0.0123 (11)	0.0184 (12)	0.0177 (12)	0.0028 (9)	−0.0007 (9)	0.0000 (10)
C10	0.0183 (12)	0.0136 (11)	0.0153 (12)	0.0021 (9)	0.0008 (9)	−0.0009 (9)
C11	0.0166 (12)	0.0153 (11)	0.0113 (11)	0.0000 (9)	−0.0001 (9)	−0.0019 (9)
C12	0.0129 (11)	0.0159 (11)	0.0113 (11)	−0.0019 (9)	−0.0002 (9)	−0.0014 (9)
C13	0.0195 (13)	0.0156 (12)	0.0169 (12)	0.0023 (10)	0.0018 (10)	0.0028 (9)
C14	0.0220 (13)	0.0119 (11)	0.0177 (12)	0.0017 (10)	0.0030 (10)	0.0008 (9)
C15	0.0104 (11)	0.0152 (11)	0.0204 (12)	−0.0015 (9)	−0.0002 (9)	0.0014 (10)
C16	0.0118 (11)	0.0125 (11)	0.0215 (13)	0.0010 (9)	0.0023 (9)	0.0024 (9)
C17	0.0141 (12)	0.0136 (11)	0.0149 (12)	0.0002 (9)	−0.0001 (9)	0.0003 (9)
C18	0.0133 (11)	0.0106 (10)	0.0164 (12)	0.0001 (9)	−0.0005 (9)	0.0022 (9)
C19	0.0113 (11)	0.0112 (11)	0.0168 (12)	0.0001 (9)	0.0009 (9)	0.0021 (9)
C20	0.0232 (14)	0.0213 (13)	0.0174 (13)	0.0005 (11)	0.0038 (10)	−0.0026 (10)
C21	0.0204 (13)	0.0207 (13)	0.0138 (12)	−0.0024 (10)	−0.0012 (10)	−0.0024 (10)

### Geometric parameters (Å, °)

**Table:**

Br1—C2	1.893 (3)	C1—C2	1.350 (4)
Br2—C9	1.891 (3)	C1—H1	0.9500
Br3—C16	1.897 (3)	C2—C3	1.412 (4)
N1—C7	1.380 (3)	C3—C4	1.384 (4)
N1—C1	1.382 (3)	C3—H3	0.9500
N1—C5	1.383 (3)	C4—C5	1.425 (3)
N2—C5	1.333 (3)	C6—C7	1.370 (4)
N2—C6	1.367 (3)	C6—H6	0.9500
N3—C4	1.358 (3)	C7—H7	0.9500
N3—H31	0.883 (10)	C8—C9	1.351 (4)
N3—H32	0.880 (10)	C8—H8	0.9500
N4—C14	1.377 (3)	C9—C10	1.418 (4)
N4—C8	1.377 (3)	C10—C11	1.369 (4)
N4—C12	1.387 (3)	C10—H10	0.9500
N5—C12	1.328 (3)	C11—C12	1.425 (3)
N5—C13	1.375 (3)	C13—C14	1.361 (4)
N6—C11	1.386 (3)	C13—H13	0.9500
N6—H61	0.875 (10)	C14—H14	0.9500
N6—H62	0.881 (10)	C15—C16	1.348 (4)
N7—C21	1.378 (3)	C15—H15	0.9500
N7—C15	1.380 (3)	C16—C17	1.408 (3)
N7—C19	1.382 (3)	C17—C18	1.385 (3)
N8—C19	1.333 (3)	C17—H17	0.9500
N8—C20	1.377 (3)	C18—C19	1.424 (3)
N9—C18	1.356 (3)	C20—C21	1.364 (4)
N9—H91	0.874 (10)	C20—H20	0.9500
N9—H92	0.876 (10)	C21—H21	0.9500
			
C7—N1—C1	130.4 (2)	C9—C8—N4	116.4 (2)
C7—N1—C5	106.6 (2)	C9—C8—H8	121.8
C1—N1—C5	123.0 (2)	N4—C8—H8	121.8
C5—N2—C6	104.5 (2)	C8—C9—C10	123.5 (2)
C4—N3—H31	120 (3)	C8—C9—Br2	118.4 (2)
C4—N3—H32	121 (3)	C10—C9—Br2	118.03 (18)
H31—N3—H32	105 (4)	C11—C10—C9	119.4 (2)
C14—N4—C8	130.4 (2)	C11—C10—H10	120.3
C14—N4—C12	106.4 (2)	C9—C10—H10	120.3
C8—N4—C12	123.2 (2)	C10—C11—N6	123.9 (2)
C12—N5—C13	104.4 (2)	C10—C11—C12	118.2 (2)
C11—N6—H61	118 (2)	N6—C11—C12	118.0 (2)
C11—N6—H62	114 (2)	N5—C12—N4	111.6 (2)
H61—N6—H62	112 (3)	N5—C12—C11	129.2 (2)
C21—N7—C15	130.2 (2)	N4—C12—C11	119.2 (2)
C21—N7—C19	106.6 (2)	C14—C13—N5	111.8 (2)
C15—N7—C19	123.2 (2)	C14—C13—H13	124.1
C19—N8—C20	104.4 (2)	N5—C13—H13	124.1
C18—N9—H91	123 (2)	C13—C14—N4	105.7 (2)
C18—N9—H92	116 (2)	C13—C14—H14	127.1
H91—N9—H92	120 (3)	N4—C14—H14	127.1
C2—C1—N1	116.3 (2)	C16—C15—N7	116.4 (2)
C2—C1—H1	121.9	C16—C15—H15	121.8
N1—C1—H1	121.9	N7—C15—H15	121.8
C1—C2—C3	124.0 (2)	C15—C16—C17	123.8 (2)
C1—C2—Br1	118.2 (2)	C15—C16—Br3	117.90 (19)
C3—C2—Br1	117.8 (2)	C17—C16—Br3	118.32 (19)
C4—C3—C2	119.3 (2)	C18—C17—C16	119.5 (2)
C4—C3—H3	120.3	C18—C17—H17	120.2
C2—C3—H3	120.3	C16—C17—H17	120.2
N3—C4—C3	124.9 (2)	N9—C18—C17	124.2 (2)
N3—C4—C5	117.6 (2)	N9—C18—C19	118.3 (2)
C3—C4—C5	117.5 (2)	C17—C18—C19	117.5 (2)
N2—C5—N1	111.6 (2)	N8—C19—N7	111.6 (2)
N2—C5—C4	128.5 (2)	N8—C19—C18	128.8 (2)
N1—C5—C4	119.8 (2)	N7—C19—C18	119.6 (2)
N2—C6—C7	112.0 (2)	C21—C20—N8	111.7 (2)
N2—C6—H6	124.0	C21—C20—H20	124.2
C7—C6—H6	124.0	N8—C20—H20	124.2
C6—C7—N1	105.3 (2)	C20—C21—N7	105.6 (2)
C6—C7—H7	127.4	C20—C21—H21	127.2
N1—C7—H7	127.4	N7—C21—H21	127.2
			
C7—N1—C1—C2	−178.7 (2)	C8—N4—C12—N5	179.0 (2)
C5—N1—C1—C2	0.7 (4)	C14—N4—C12—C11	176.1 (2)
N1—C1—C2—C3	1.9 (4)	C8—N4—C12—C11	−3.6 (4)
N1—C1—C2—Br1	−177.80 (17)	C10—C11—C12—N5	179.6 (2)
C1—C2—C3—C4	−2.1 (4)	N6—C11—C12—N5	−0.2 (4)
Br1—C2—C3—C4	177.53 (18)	C10—C11—C12—N4	2.7 (3)
C2—C3—C4—N3	−178.6 (2)	N6—C11—C12—N4	−177.1 (2)
C2—C3—C4—C5	−0.2 (4)	C12—N5—C13—C14	−0.2 (3)
C6—N2—C5—N1	0.7 (3)	N5—C13—C14—N4	−0.6 (3)
C6—N2—C5—C4	−176.3 (2)	C8—N4—C14—C13	−179.2 (2)
C7—N1—C5—N2	−0.6 (3)	C12—N4—C14—C13	1.1 (3)
C1—N1—C5—N2	179.8 (2)	C21—N7—C15—C16	178.8 (2)
C7—N1—C5—C4	176.6 (2)	C19—N7—C15—C16	−1.5 (4)
C1—N1—C5—C4	−3.0 (4)	N7—C15—C16—C17	0.1 (4)
N3—C4—C5—N2	−2.2 (4)	N7—C15—C16—Br3	−179.89 (17)
C3—C4—C5—N2	179.3 (2)	C15—C16—C17—C18	1.8 (4)
N3—C4—C5—N1	−178.9 (2)	Br3—C16—C17—C18	−178.21 (18)
C3—C4—C5—N1	2.6 (3)	C16—C17—C18—N9	176.5 (2)
C5—N2—C6—C7	−0.5 (3)	C16—C17—C18—C19	−2.3 (3)
N2—C6—C7—N1	0.1 (3)	C20—N8—C19—N7	−0.1 (3)
C1—N1—C7—C6	179.8 (2)	C20—N8—C19—C18	179.5 (2)
C5—N1—C7—C6	0.3 (3)	C21—N7—C19—N8	0.3 (3)
C14—N4—C8—C9	−178.1 (2)	C15—N7—C19—N8	−179.5 (2)
C12—N4—C8—C9	1.6 (4)	C21—N7—C19—C18	−179.3 (2)
N4—C8—C9—C10	1.3 (4)	C15—N7—C19—C18	0.9 (4)
N4—C8—C9—Br2	178.65 (17)	N9—C18—C19—N8	2.6 (4)
C8—C9—C10—C11	−2.0 (4)	C17—C18—C19—N8	−178.5 (2)
Br2—C9—C10—C11	−179.38 (19)	N9—C18—C19—N7	−177.8 (2)
C9—C10—C11—N6	179.7 (2)	C17—C18—C19—N7	1.0 (3)
C9—C10—C11—C12	−0.1 (4)	C19—N8—C20—C21	−0.2 (3)
C13—N5—C12—N4	0.9 (3)	N8—C20—C21—N7	0.4 (3)
C13—N5—C12—C11	−176.2 (2)	C15—N7—C21—C20	179.3 (2)
C14—N4—C12—N5	−1.3 (3)	C19—N7—C21—C20	−0.4 (3)

### Hydrogen-bond geometry (Å, °)

**Table:**

*D*—H···*A*	*D*—H	H···*A*	*D*···*A*	*D*—H···*A*
N3—H31···N5	0.88 (1)	2.16 (1)	3.034 (3)	169 (4)
N6—H61···N2	0.88 (1)	2.23 (1)	3.094 (3)	168 (3)
N9—H91···N8^i^	0.87 (1)	2.27 (2)	3.091 (3)	158 (3)
N9—H92···N6^ii^	0.88 (1)	2.27 (1)	3.143 (3)	177 (3)

Symmetry codes: (i) −*x*+1, −*y*+1, −*z*; (ii) *x*, *y*, *z*−1.
